# Compaction of quasi-one-dimensional elastoplastic materials

**DOI:** 10.1038/ncomms15568

**Published:** 2017-06-06

**Authors:** M. Reza Shaebani, Javad Najafi, Ali Farnudi, Daniel Bonn, Mehdi Habibi

**Affiliations:** 1 Department of Theoretical Physics, Saarland University, 66041 Saarbrücken, Germany; 2 Department of Experimental Physics, Saarland University, 66041 Saarbrücken, Germany; 3 Department of Physics, Institute for Advanced Studies in Basic Sciences, Zanjan 45195, Iran; 4 Van der Waals-Zeeman Institute, University of Amsterdam, 1098 XH Amsterdam, The Netherlands; 5 Present address: Laboratory of Physics and Physical Chemistry of Foods, Wageningen University, Wageningen, The Netherlands

## Abstract

Insight into crumpling or compaction of one-dimensional objects is important for understanding biopolymer packaging and designing innovative technological devices. By compacting various types of wires in rigid confinements and characterizing the morphology of the resulting crumpled structures, here, we report how friction, plasticity and torsion enhance disorder, leading to a transition from coiled to folded morphologies. In the latter case, where folding dominates the crumpling process, we find that reducing the relative wire thickness counter-intuitively causes the maximum packing density to decrease. The segment size distribution gradually becomes more asymmetric during compaction, reflecting an increase of spatial correlations. We introduce a self-avoiding random walk model and verify that the cumulative injected wire length follows a universal dependence on segment size, allowing for the prediction of the efficiency of compaction as a function of material properties, container size and injection force.

## Abstract (web summary)

Principles underlying crumpling of one-dimensional objects may be relevant to both biomolecular processes and to design of mechanical devices. By compacting various wires under rigid confinement and modelling observed geometric features, the authors show how friction, plasticity and torsion enhance disorder and lead to a transition from coiled to folded geometries.

Compaction of slender objects in confined geometries is ubiquitous in nature. Perhaps the most important example is DNA packaging in viral and bacteriophage capsids and cell nuclei^1^,^2^,^3^,^4^,^5^. Other pertinent examples are the folding of insect wings in cocoons^6^, and flower or plant leaves in buds^7^. The process of compaction may result in complex morphologies depending on the applied forces and constraints^8^. Recent numerical studies^9^,^10^,^11^,^12^,^13^ showed that the space-filling properties of two-dimensional (2D) crumpled sheets are influenced by parameters such as self-avoidance and plasticity–ingredients that are difficult to disentangle in experiments. Self-avoidance alters the hierarchical nature of the compaction process and induces stronger self-correlations as the compression increases^13^,^14^. Thus, considering the structural evolution is key for understanding the efficiency of *in vitro* compaction.

Aiming to provide quantitative insights into the role of self-avoidance, we turn to one-dimensional (1D) wires. Due to its very nonlinear nature it is easier to study 1D systems than the more complicated crumpling process in 2D sheets^13^,^15^,^16^,^17^. For 1D-compaction, how the morphology of crumpled objects develops is of particular importance in technological and biological applications as, for example, in endovascular coiling treatment of cerebral aneurysms^18^ or in packing of DNA^19^.

When compacting elastic low-frictional wires with a high bending rigidity in confined geometries in such a way that the internal torsion is released, highly ordered structures with distinctly oriented subdomains of parallel coils form (Fig. 1). However, with increasing friction^20^ or plasticity^9^, or by accumulating torsion during the packing process^21^,^22^, disordered structures emerge where the contribution of folds or bends in the morphology is more pronounced. For example, by introducing the number of segments as the order parameter, it has been recently shown that a sharp transition from ordered (coiled) to disordered (folded) structures occurs as the friction increases^20^. In disordered morphologies, the compaction efficiency is controlled to a large extent by spatial exclusion effects, which continuously evolve in the course of compaction. Hence, unravelling the mechanisms that govern the evolution of self-avoidance is crucial to achieve an efficient compaction.

Here, we study the morphologies of wires packed into rigid spherical containers and find that the maximum packing density in disordered structures decreases with reduced thickness of the wire (or, equivalently, increasing the container size). To elaborate on the underlying mechanisms leading to this peculiar behaviour, we isolate the influence of self-avoidance by focusing on the compaction of plastic frictional wires, where folding is dominant in the resulting structure. By following the morphological evolution, a gradual crossover from random to correlated folding events is observed due to spatial exclusion effects. We propose that the compaction can be considered as a confined self-avoiding random walk (SAW). In such far-from-equilibrium processes, the imposed constraints and initial conditions do not uniquely determine the final crumpled state. Instead, there is an ensemble of admissible configurations, from which some structural properties of the system can be derived. We introduce a SAW sampling method that successfully accounts for the time evolution of the wire segment length. We thus present a more complete understanding of the compaction: the maximum length of the injected wire can be estimated from the geometry and imposed constraints for a given set of material parameters.

## Results

### Universal phase diagram for 1D crumpling

**Figure 1:**
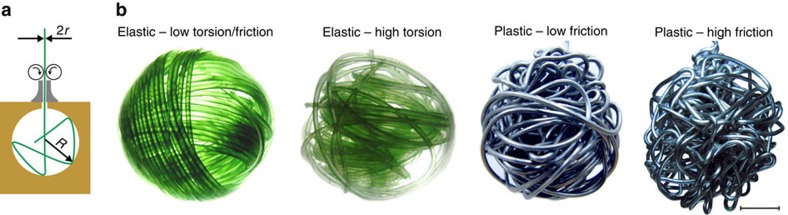
Experimental set-up and examples of distinct morphologies. (**a**) Cross-sectional view of the experimental set-up used to compact 1D wires in a spherical rigid container. (**b**) Distinct morphologies obtained by compacting wires with different material properties introduced in the Methods section. Scale bar, 1 cm.

**Figure 2:**
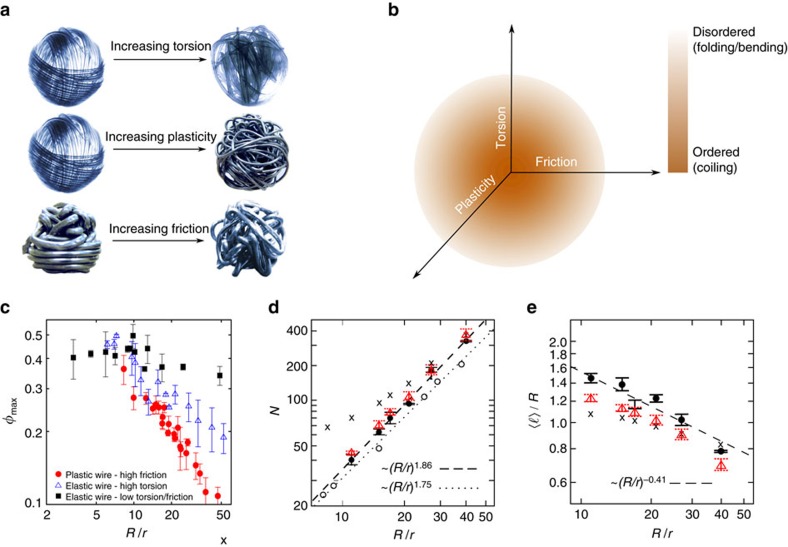
Morphological phase diagram and scaling of geometrical characteristics. (**a**) Examples of morphological differences upon increasing key material parameters. (**b**) Schematic morphological phase diagram in the space spanned by torsion, friction and plasticity. (**c**) The maximum packing density *φ*_max_ versus the relative system size 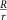 (parameters defined in Fig. 1a). (**d**,**e**) Scaling of the number of bends *N* (**d**), and the dimensionless mean segment size 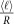 (**e**) versus the effective system size 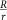, for plastic frictional wires. The filled circles (open triangles) indicate experimental (simulation) results. The dashed lines are power-law fits to the experimental data. The crosses denote the simulation results obtained when assuming that the packing fraction is the influential parameter on the self-avoidance effects. The open circles in (**d**) show the experimental results obtained from the high frictional set-up. Error bars correspond to s.d. of five separate measurements.

We first consider the packing of elastic low-frictional wires with a high bending rigidity in rigid spherical containers. When the wire is allowed to axially rotate at the injection point to release the torsion during the packing process, highly ordered coils form as the wire relaxes towards a global minimum energy. By hindering the release of torsion, the wire buckles more frequently to free elastic energy. Hence, the packing process becomes less ordered^21^, leading to warped structures similar to those obtained numerically for compaction of DNA molecules in phage capsids^4^ (see Figs 1 and 2a). The disorder is also enhanced by friction, which causes the wire to resist against sliding and to randomly bend due to local constraints^20^. Another property which obviously affects the morphology is the degree of plasticity of the wire. While the bending rigidity of the plastic wires can be quite high, their yield stress is relatively low, leading to structures with rather straight segments and sharp turnings. Upon increasing plasticity (that is, lowering the yield stress), the irreversible deformations of wire increase the disorder of the crumpled configuration. One can map out a qualitative phase diagram for the morphological evolution of the resulting crumpled structures in the space of wire properties (friction, torsion and plasticity), as depicted in Fig. 2b. More generally, disordered structures can be generated in diverse ways by tuning the wire or container properties. The morphological phase space indeed contains additional degrees of freedom associated with container properties, such as its flexibility^20^, shape^23^ or the degree of confinement imposed by it (characterized by the container size *R* relative to the radius of gyration *R*_g_ of the crumpled structure and also to the persistence length *λ* of the elastic wire). For example, a biopolymer coils itself inside the cage if *λ* is comparable to *R* (for example, in packing of DNA in icosahedral bacteriophages), while for weak confinement, that is, 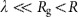, the biopolymer chain (such as chromatin) has a relatively low bending stiffness and behaves as a SAW without ‘feeling' the boundaries. For *λ* values in between, the morphological evolution during the crumpling process is complicated due to varying combined effects of self-avoidance and interactions with boundaries^19^,^24^. It has been also shown that a transition from coiled to disordered configurations occur, as the accessible space reduces during the compaction of elastic rods^25^.

To compare the compaction efficiencies, we measure the packing density once the injection of wire eventually stops. Indeed, the value obtained for this maximum packing density, *φ*_max_, depends on the wire radius *r*, the container size *R* and the insertion force. We measure this quantity for a given insertion force and for different combinations of inserted wire radius *r* and container radius *R*. When plotting *φ*_max_ versus the non-dimensional system size 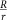 for the coiled compact morphologies of low-plasticity, low-friction, low-torsion wires (Fig. 2c), a plateau at small 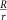 followed by a weak decrease at larger values of 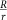 is observed. It was shown with geometrical arguments^22^ that *φ*_max_ slightly decays with 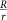 for a purely coiled structure in a spherical container. Note that the very inner core of the structure practically becomes disordered as the accessible space reduces and its shape becomes more irregular (which makes the formation of coils more difficult). This disordered core (with a possible 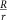-dependent size) can also contribute to the weak decay of *φ*_max_ versus 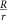. It has been shown that the packing fraction decreases with increasing disorder in packings of elastic wire^26^.

When increasing plasticity, friction and/or torsion, resulting in the formation of folds and bends, the data collapse onto curves following a power-law 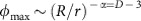, with *D* being the fractal dimension^27^,^28^. The slope of the curve depends on the degree of disorder. For example, lubricating the inner wall of the container with silicon oil leads to the formation of highly ordered coils at the outer layer of crumpled plastic wires, which results in a mixed coiled-folded structure with 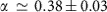. A similar exponent is obtained for the compaction of elastic torsional wires where coils and bends coexist. The steepest descent is observed for crumpling of plastic wires at high friction, where coils are absent and folding is the dominant process (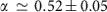). While the very weak system-size dependence of efficient compaction in ordered (coiled) structures is understandable, the behaviour of disordered morphologies is counter-intuitive, as one would expect that relatively thinner wires more flexibly fill a given container, leading to a higher compaction efficiency. A similar trend for the dependence of packing density on the relative system size was reported in experiments on DNA packaging in viral capsids^29^, revealing that in spite of the huge differences in length scales of the two systems, the maximum packing densities behave similarly in the presence of disorder. Self-avoidance inside a confinement can explain the peculiar behaviour of *φ*_max_ versus 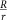 via a mean-field interpretation, assuming that the self-avoidance energy originates mainly from the homogeneously distributed binary contacts between the wires (whose density nearly grows as the square of the packing fraction), and also supposing that the local radius of curvature of the confinement is comparable to the container size and varies slowly. When balancing the confining energy and self-avoidance^30^,^31^,^32^, the lowest (harmonic) approximation of the confining energy yields an energy density of the order of 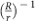 while the self-avoidance energy density is proportional to 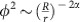. By equalizing these energy densities we obtain 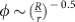.

### Segment size statistics

To better understand the influence of disorder, we choose plastic frictional wires to avoid ordered coils and create the highest possible disorder in the crumpled structure. After compacting the wires, we open the moulds and investigate the resulting compacted structures by analyzing the folding statistics. The points of folding were often determined by sharp changes of wire orientation. If they were not easily distinguishable, then a minimum threshold of 90° for the turning angle of the wire, and a maximum threshold of 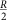 for the radius of curvature were imposed. We cut the wire at each of the folding points, straighten the segments and measure their length. Straightening of the curved segments rarely allows for segment lengths 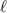 longer than the container diameter, but we checked that the maximum segment length 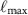 remains smaller than *πR* in the absence of coils. We preserve the order of the wire segments and average the results over five realizations for each value of *R*/*r* to obtain the sequence of the segment lengths 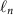.

A key observation is the scaling of the total number of segments *N* with the effective system size 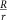. Similar scaling laws were reported for 2D packings of wires^27^,^30^,^33^. As shown in Fig. 2d, a power-law relation of the form


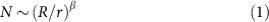


holds with *β*=1.86±0.03 for smooth wire and container with wire–wire and container-wire friction coefficients 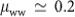 and 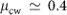, respectively. The exponent can be understood by considering the wire crumpling process as a SAW in confinement. For comparison, for the number of steps on a cubic lattice in an ordinary random walk *β* equals 2, while for SAW 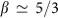 (ref. 34). The fact that we find an exponent in between these two values can be understood because there is a gradual evolution of spatial correlations over the course of crumpling (see below), thus, the exponent continuously decreases from 2. However, the wires can slide over each other due to the finite friction so that the self-avoidance constraint is only partially fulfilled. For comparison, we repeated the experiment by roughening the plastic wires and the inner surface of the moulds to increase the friction coefficients to 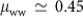 and 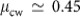. The considerable change in the wire–wire friction resulted in a smaller exponent *β*=1.75±0.05 which is closer to the pure self-avoidance limit (see Fig. 2d). From the scaling of *φ*_max_ and *N* with 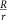 one expects that the normalized mean segment size 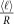 follows 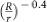, as confirmed by the experimental results in Fig. 2e.

### Evolution of spatial correlations

**Figure 3:**
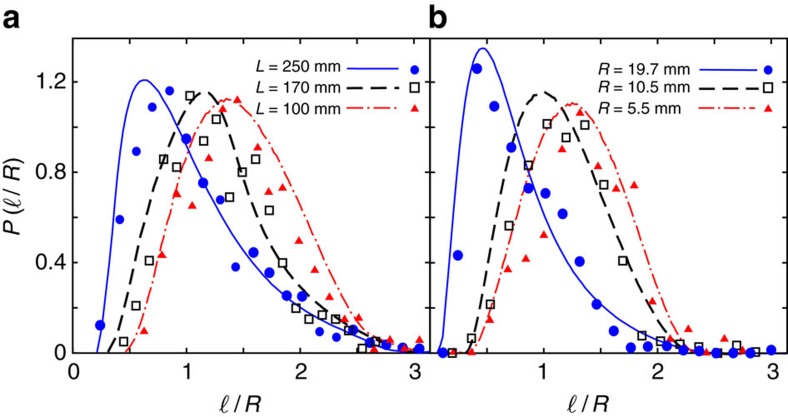
Probability distribution of the normalized segment size. The symbols (lines) represent the experimental (simulation) results for *r*=0.5 mm. (**a**) results obtained by injecting wires of different total length *L* into a container with radius *R*=14.5 mm. (**b**) results of injecting the maximum possible length of wire (when applying the insertion force of nearly *F*=100 N) in different container sizes *R*.

At earlier stages of the crumpling process in a given mould, the injected wire proceeds in the container without interacting with the accumulated wire. Assuming that the plastic wire bends at a random point between the injecting hole and a contact point at the container surface, the resulting segment length 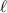 is a random variable, symmetrically distributed between 0 and the maximum possible segment length 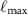. By increasing the total length *L* of accumulated wire, spatial exclusion effects grow and the injected wire cannot easily proceed through the sphere without touching the crumpled structure. Hence, long segments gradually become less probable and the probability distribution 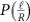 of the normalized segment size becomes more asymmetric due to relatively large populations of smaller segments (see Fig. 3a). When comparing the final structures (that is, those obtained when the injection of wire stops), we interestingly find that for larger values of 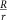 the segment size distribution 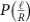 is more asymmetric and shifts towards smaller segment sizes (Fig. 3b). This behaviour similarly indicates the growth of spatial exclusion effects with increasing 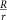. Note that the initial segment sizes 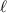 are only determined by the container size *R* in all containers, however, 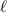 gradually decreases as the spatial exclusion effects grow. The effect is more pronounced for larger spheres as the crumpling process continues further.

### Self-avoiding random walk model

We argue that the strength of self-avoidance effects is indeed captured by the total length *L* of the injected wire, rather than the total volume excluded by it (that is, the packing fraction). The exclusion effect that an inserted rod-like object experiences inside a crumpled structure is effectively determined by the projection of the crumpled wire on a plane perpendicular to the direction of insertion. Therefore, the total length and the thickness of the crumpled wire are expected to be the influential parameters. However, the circular cross-section of wires reduces the contact area between the touching wires and, thus, the effective frictional force between them. As a result, the self-avoidance effects are not proportionally increasing with the wire thickness (that is, *r*). We conclude that the entire contribution to the spatial exclusion constraint can be attributed to the length of wire, reflected in the dimensionless quantity *λ*=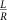 which grows as 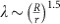 (while *φ* decreases as 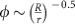). In the following, we simulate the folding process as a SAW of the wire inside the confinement. While the existing SAW algorithms mainly follow stochastic Markovian dynamics to sample the ensemble of trajectories on regular lattices, here we propose an alternative approach which accounts for the time evolution of the step size 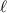. We suppose that the strength of self-avoidance effects after the *n*-th segment is mainly controlled by the length *L*_*n*_ of the inserted wire, that is, the larger is the parameter 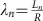, the smaller is the success probability for the segment *n*+1 to be a long one. The size 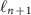 of the next segment is obtained via the following algorithm: a trial segment size 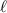 is chosen randomly within 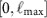, with 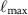 being the maximum segment length obtained in experiments for a given value of *R*. The proposed 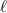 is accepted according to a Metropolis-like criterion with probability


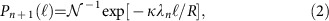


where 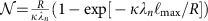 is the normalization factor. The coefficient *κ* depends on wire properties and is treated as a free parameter to take into account the partial fulfillment of the self-avoidance constraint due to sliding of the wires. While the exponents are not affected by the choice of *κ*, by fitting it we can quantitatively reproduce the experimental data. For the sake of simplicity, here we used a single averaged value of *κ* to reproduce all the experimental data. In the case of rejection, a new 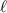 is tried. Finally, the cumulative length is updated as 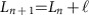 before starting the next step. Equation (2) assumes an exponentially lower acceptance chance for larger trial lengths 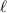. Moreover, the acceptance probability decreases with increasing *λ*_*n*_, as the self-avoidance effects become more pronounced. The method samples the segment-length landscape according to a Boltzmann-like distribution. Initially, the wire walks in free space (*λ*_*n*_=0), thus, 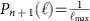 independently of the trial length 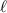. However, larger values of 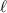 become gradually less probable with increasing *λ*_*n*_. We perform extensive Monte Carlo simulations by adjusting 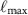 and the threshold value of *φ* to the experimental data. The results shown in Figs 2 and 3 are in remarkable agreement with experiments. We checked that the power-law scalings cannot be reproduced when replacing *λ*_*n*_ with *φ*_*n*_ in equation (2) (see Fig. 2d,e). Notably, the numerical predictions for the total wire length *L* differ less than 4% from the experimental values for all system sizes.

**Figure 4:**
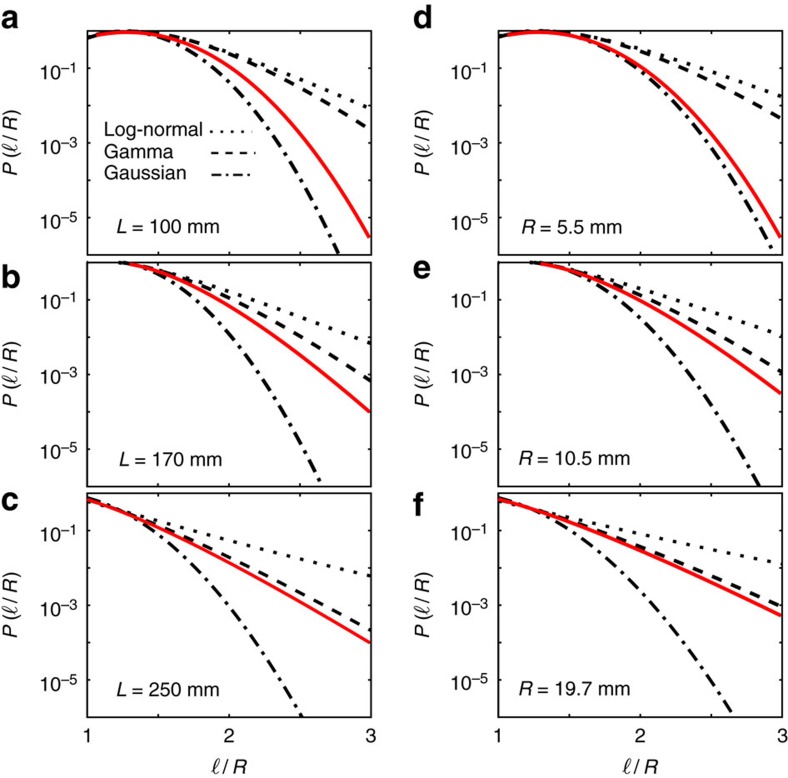
Evolution of the tail of the segment-size distribution. The results obtained from the simulations for *r*=0.5 mm. (**a**–**c**) Comparison between the tail of 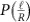 for different values of the total length *L* of the injected wire in a container with radius *R*=14.5 mm. (**d**–**f**) A similar comparison when the maximum possible length of wire is injected in different container sizes *R*. The solid lines denote the simulation results, and the dotted, dashed, and dashed-dotted guidelines represent, respectively, the log-normal 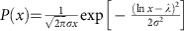, gamma 
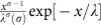
 and Gaussian 
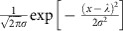
 distributions, plotted with the same mean and variance as the corresponding experimental data. In the gamma distribution, Γ(*σ*) denotes the gamma function evaluated at *σ*.

In Fig. 4 we take a closer look at the tail of 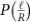 obtained from the numerical simulations. While Gaussian function represents the distribution of random uncorrelated data, gamma and log-normal functions are respectively associated with random events in the presence of self-correlations and those that occur hierarchically^8^,^11^,^13^,^35^,^36^,^37^. Since the noisy data of the experimental tail prevents any conclusive statement on the tail behaviour of 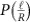, we plot these three functions (with the same mean and variance as the experimental data) and use them as guidelines to demonstrate the trend of the tail behaviour obtained from the numerical simulations. We investigate the evolution of the tail during the injection of wire in a given container, and also compare the tails when the maximum possible length of wire is injected in different container sizes. As shown in Fig. 4, the tail is better captured by the Gaussian for small 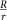 or at the early stages of crumpling, while there is a gradual crossover towards the Gamma distribution, either by increasing the container size or by increasing the length of the injected wire. Thus, self-correlations are the dominant underlying mechanism here. A hierarchical folding mechanism is expected to cause a rather stable log-normal distribution tail over all timescales, thus, the evolution of the tail is in favour of evolving correlated events. It has been previously shown numerically^13^ and by compacting of rods in 2D experiments^14^, that self-avoidance alters the hierarchical nature of crumpling at high compression and induces self-correlations.

**Figure 5:**
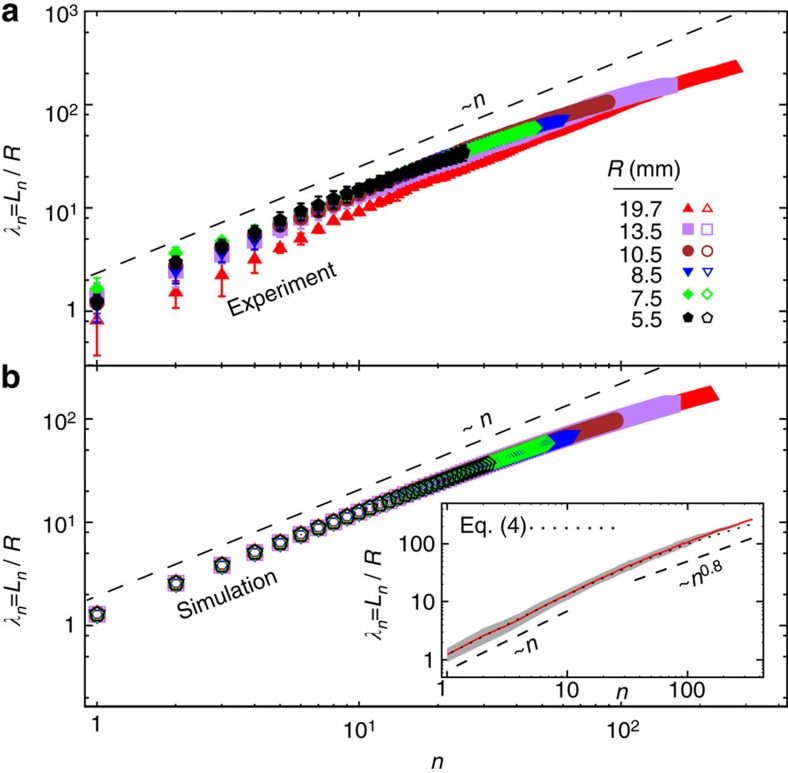
Cumulative length *L*_*n*_ of inserted wire after the *n*-th segment. (**a**) experimental results, (**b**) simulation results. Inset shows *L*_*n*_/*R* averaged over all experiments (red solid line) and its s.d. (grey shaded area). The dotted line is obtained from equation (4).

The cumulative length *L*_*n*_ of the inserted wire after the *n*-th segment qualitatively collapses onto a master curve for different values of 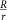 (Fig. 5). The segments are initially independent of each other and *L*_*n*_/*R* grows linearly with *n*. The steps however become more correlated with increasing *n*, leading to a slower growth of *L*_*n*_/*R*. A similar reduction of the slope has been recently observed for motor-driven viral packaging^38^. From the scaling of *φ* and *N* with 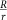, one obtains the asymptotic scaling 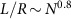, which is consistent with experiments [see Fig. 5(inset)]. Starting from 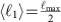, the mean segment size at next steps can be estimated in terms of *L*_*n*_ as


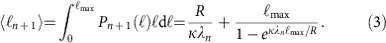


Hence, we obtain the following recursive analytical expression for the injected length of wire after the *n*-th segment


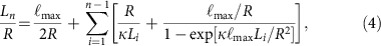


in excellent agreement with the data as shown in Fig. 5(inset).

### Buckling threshold

**Figure 6:**
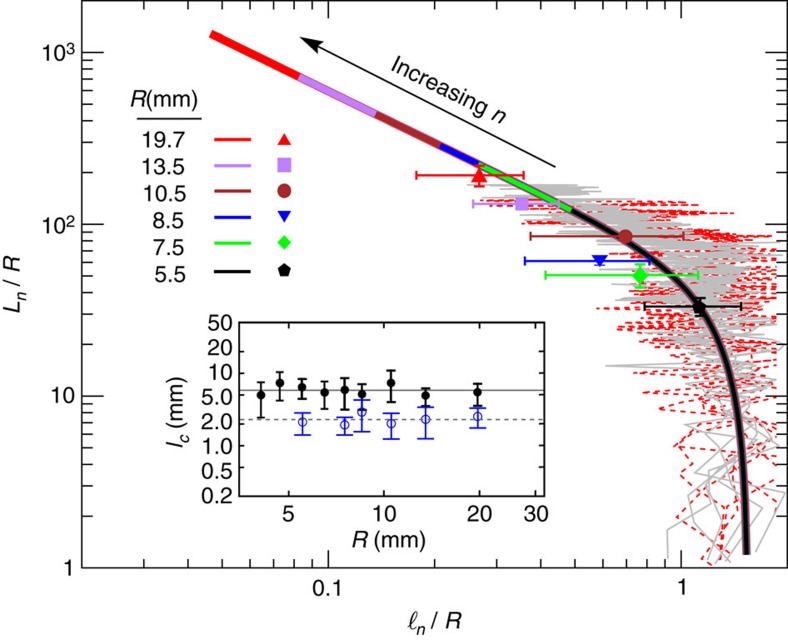
Universal filling mechanism. Collapse of *L*_*n*_/*R* versus the scaled segment length 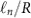 is shown for different values of 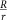 in experiments (background grey lines) and simulations (thick coloured lines). The symbols show the experimental cutoff values 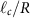 for *r*=0.5 mm. The red dashed lines indicate the experimental results when injecting wires of different total length *L*≈100, 170 or 250 cm into a container with radius *R*=14.5 mm. Inset shows cutoff segment length 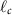 versus the container radius *R* for *r*=0.3 mm (open circles) and *r*=0.5 mm (filled circles). The horizontal lines show the average values 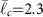 and 5.8 mm. Error bars correspond to s.d. of five separate experimental measurements.

The inset of Fig. 6 shows that the injection of wire eventually stops at a cutoff segment length 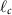 which is independent of *R* for a given *r*. It follows that the process stops when the segment size becomes so small that the applied feeding force *F* exceeds the maximum power of the motor. The minimum threshold length can be obtained from the Euler buckling theory as 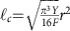 so that 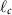 is determined by the insertion force and wire properties (Young's modulus *Y*) and is independent of *R*. For *r*=0.3 mm (0.5 mm) and a maximum insertion force of nearly *F*=100 N in our set-up, we obtain 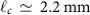 (6.0 mm), close to the experimental values of 2.3 and 5.8 mm [Fig. 6(inset)].

Figure 6 interestingly evidences a universal filling mechanism independent of *R*. The normalized cumulative wire length *L*_*n*_/*R* vs. the normalized length of the *n*-th segment 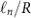 collapses onto a universal curve for different values of 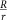. As the wire injection continues, 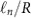 gradually decreases until it reaches the minimum threshold value 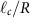, where the process eventually stops. For a given value of *r*, 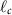 is independent of *R*, thus, 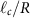 decreases (that is, shifts to the left in Fig. 6) with increasing *R*. Consequently, the cutoff number of the steps at which the process stops increases with *R*. By calculating 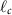 from the insertion force and wire properties, one can predict the total length of crumpled wire for different *R*. The data collapse is also obtained when injecting wires of different total length *L* into a given container (dashed lines in Fig. 6). Indeed, there is no significant difference between the sequence of the segment sizes 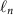 for different total lengths of the injected wire. This shows that the previously formed segments are not considerably affected during the compaction process, evidencing that the hierarchical folding events rarely happen.

In summary, we reported the compaction of 1D objects in spherical containers and showed how the morphology evolves from ordered (coiling) to disordered (folding and bending) structures in the phase space spanned by friction, torsion and plasticity. The disorder reduces the compaction efficiency and causes a nontrivial system-size dependence, which is explained by SAWs in confined geometries. Monitoring the evolution of segment-length distributions in highly disordered structures of plastic frictional wires showed that the compaction process is correlated: the longer the injected wire, the stronger the spatial exclusion effects leading to shorter segments. The self-avoidance constraint is only partially fulfilled due to sliding of the wires, leading to an exponent *β* slightly larger than 5/3 as reported for SAWs. Our results provide new insight into underlying mechanisms of crumpling beyond the simple hierarchical description of the process, which also helps to better understand the reverse processes, for example, viral DNA ejection^39^ and unpacking of crumpled wires^40^. While more detailed morphological information can be obtained via Discrete-Element Method (DEM) simulations, these are however computationally expensive. Our proposed sampling method opens the door to relatively simple Monte Carlo simulations of SAWs inside arbitrary confinements to obtain some of the macroscopic quantities of interest for example, the arbitrary moments of segment size distribution.

## Methods

### Experimental set-up

The experimental set-up consists of a rigid hollow spherical container of inner radius *R* with a small hole to insert the wire (see Fig. 1). Several transparent polymeric moulds with radii *R*∈[4,30 mm] were used. A small nozzle and two counterrotating rollers were attached to the injecting hole to facilitate the control of the insertion speed.

### Material properties

As a model elastoplastic material, we chose solder wire Sn_60_Pb_40_ with Young's modulus *Y*≈30 GPa and yield stress *σ*≈28 MPa. For the elastic wire experiments, we mainly used fishing line with Young's modulus *Y*=2.00±0.01 GPa (obtained experimentally by tensile tests). Moreover, elastic silicon wires and cotton threads with relatively lower Young's moduli *Y*≈5.0 and 0.8 MPa were also used. The wire–wire and container-wire friction coefficients, using smooth wires and container walls, were *μ*_ww_=0.20±0.02 and *μ*_cw_=0.40±0.02, respectively. By roughening the plastic wires and the inner surface of the polymeric moulds with sandpaper, we obtained higher friction coefficients *μ*_ww_=0.45±0.02 and *μ*_cw_=0.45±0.02. We also used smooth lubricated plastic wires and inner surfaces of the moulds to lower the friction coefficients, leading to *μ*_ww_=0.12±0.02 and *μ*_cw_=0.18±0.02. The lubrication was done with silicon oil.

### Insertion process and imaging

We inserted wires of radius *r*=0.4, 0.5, 0.6, 0.8 or 1.4 mm into the moulds with a slow feeding speed of about 1 mm s^−1^ to avoid inertial effects. We checked that the results are independent of the feeding speed in the quasi-static compaction regime. The insertion process continued with the constant speed until the insertion force exceeded the power threshold of the motor and the wire buckled outside of the container. The final plastic-wire structure preserves its shape after opening the mould allowing for a detailed analysis of morphological changes, which can be considered as plastic deformations. In the low-torsion elastic set-up, we allowed axial rotation of the wire between the nozzle and the sphere. The images presented in Figs 1 and 2 were taken by a camera with pixel resolution of 70 μm. Before opening the moulds, we filled them with a transparent gel in the case of elastic wires to preserve the shape of the final structure.

### Data availability

The data that support the findings of this work are available from the corresponding authors on request.

## Additional information

**How to cite this article:** Shaebani, M. R. *et al*. Compaction of quasi-one-dimensional elastoplastic materials. *Nat. Commun.* **8**, 15568 doi: 10.1038/ncomms15568 (2017).

**Publisher's note**: Springer Nature remains neutral with regard to jurisdictional claims in published maps and institutional affiliations.
